# A New Surgical Method of U-Shaped Myometrial Excavation and Modified Suture Approach with Uterus Preservation for Diffuse Adenomyosis

**DOI:** 10.1155/2018/1657237

**Published:** 2018-07-09

**Authors:** Xie Jun-Min, Zhu Kun-Peng, Zhao Yin-Kai, Zhang Ya-Qin, Fan Xiao-Fan, Zhu Xiao-Yu, Wei Li, Wang Bin

**Affiliations:** ^1^Department of Obstetrics and Gynecology, Zhengzhou Hua-Shan Hospital, Zhengzhou 450000, Henan, China; ^2^Department of Orthopaedic Surgery, Shanghai Tenth People's Hospital, Tongji University School of Medicine, Shanghai 200072, China; ^3^Institute of Bone Tumor Affiliated to Tongji University, Shanghai 200072, China; ^4^Department of Obstetrics and gynecology, The Third Affiliated Hospital of Zhengzhou University, Zhengzhou 450000, Henan, China; ^5^Department of Obstetrics and Gynecology, Henan Provincial People's Hospital, Zhengzhou 450003, Henan, China

## Abstract

**Objective:**

To evaluate the feasibility, safety, and efficacy of a new surgical method of U-shaped myometrial excavation and modified suture approach with uterus preservation for diffuse adenomyosis.

**Methods:**

From January 2012 to December 2014, 198 patients with diffuse adenomyosis were surgically treated using this novel procedure in Zhengzhou Hua-Shan Hospital. Degree of dysmenorrhea, menstrual blood volume, serum CA 125, and uterine size before and at 1 month, 3 months, 6 months, 12 months, and 24 months after surgery were compared.

**Results:**

Postoperatively, VAS score of dysmenorrhea, menstrual blood volume, serum CA 125 level, and uterine size significantly decreased at 1 month, 3 months, 6 months, 12 months, and 24 months from presurgical levels (all p < .001), but there were no differences at the follow-up time points. Two patients recurred at 18 months and 23 months after surgery, but both recovered after repeat surgery. Interestingly, 2 other patients recrudesced at 10 months and 12 months after surgery. In addition, only one patient was found to have a postoperative anaemia with fever, conservatively managed without surgery.

**Conclusion:**

U-shaped myometrial excavation and modified suture approach with uterus preservation is a safe and feasible surgical approach to treat diffuse adenomyosis, with favourable outcomes.

## 1. Introduction

Adenomyosis is a heterogeneous gynaecological disease, characterized by the presence of ectopic endometrial glands and mesenchymal cells that grow into the myometrium and form partly or diffusely dilated islets [1]. Adenomyosis primarily occurs in postpartum women; however, its age of onset has been trending younger in recent years [2]. Dysmenorrhea, menorrhagia, severe anaemia, and infertility are all clinical manifestation [3]. Because of these characteristics, it is called the “living cancer.”

It is challenging for both patients and physicians to choose the best therapeutic regimen, because the aetiology and pathogenesis of adenomyosis remain unclear and there is no accurate diagnostic imaging standard [4]. The current standard treatment for adenomyosis is hysterectomy [5]. For women who have a fertility requirement, adenomyosis lesion resection with uterus preservation is the only available effective treatment [6]. However, it is too difficult to treat those patients with diffuse adenomyosis using lesion resection owing to the invisible clear boundaries [1].

Herein, we report a new surgical method for women suffering from diffuse adenomyosis but who wish to preserve their uterus. The key to this new surgical technique is combining U-shaped myometrial excavation with a modified suture approach. Our aim in this study was to present our experience regarding the feasibility, safety, and efficacy of this novel surgical approach of uterus preservation for diffuse adenomyosis surgery.

## 2. Materials and Methods

### 2.1. Participants

This retrospective study was performed at the Zhengzhou Hua-Shan Hospital from patients undergoing surgery between January, 2012, and December, 2014. Patients meeting criteria were enrolled in the study. China Clinical Trials Registry Number is ChiCTR1800016740.

Inclusion criteria were as follows: (1) dysmenorrhea with progressive menorrhagia; (2) diffuse adenomyosis preoperatively preliminary diagnosed by vaginal ultrasound B and MRI; (3) request to keep uterus or having fertility requirements; (4) insufficient response to drugs including oral contraceptives and GnRHa treatment; (5) ineffective or unsuccessful response to levonorgestrel (Mirena); (6) inability to give birth by themselves or after assisted reproductive technology; (7) strong requirement to conduct the U-shaped surgery to preserve the uterus without contraindications; and (8) signing the informed consent form.

All patients were diagnosed on the basis of medical history, physical examination, and the results of transvaginal ultrasound (TVS) and magnetic resonance imaging (MRI). All interpretations of TVS and MRI for the diagnosis of diffuse adenomyosis were performed by one of the authors together with several radiologists and ultrasound section physicians. Each patient was sufficiently informed regarding all aspects of management before surgery, and written consent was obtained.

### 2.2. Data Collection

Patient information, including sociodemographic characteristics, final postoperative pathological diagnosis, and preoperative, intraoperative and postoperative parameters were collected. A visual analogue scale (VAS) ranging from 0 (no pain) to 10 (severe pain, intolerability) was used to evaluate the degree of dysmenorrhea. Menstrual blood loss was assessed using the Mansfield-Voda-Jorgensen (MVJ) menstrual bleeding scale, ranging from 1 (spotting) to 6 (gushing). When MVJ score was equal to or greater than 5, it was considered hypermenorrhoea. The uterine size was measured by TVS [uterine volume (cm^3^) = length (cm) × width (cm) × height (cm) × 0.5236] [7]. The serum CA 125 levels were determined by enzyme-linked immunosorbent assay (ELISA) using a sandwich ELISA kit according to the manufacturer's instructions (normal CA 125 level, ⩽35 U/mL).

### 2.3. Surgical Procedure

In general, surgery was performed as soon as possible after menstruation, because the endometrium was thinnest at this time. The operation was performed under epidural anaesthesia or general anaesthesia, and all were performed via laparotomy. A lower abdominal transverse or longitudinal incision was made, and the length of the incision depended on the size of the uterus. Then, the uterus and both fallopian tubes were exposed. In this procedure, both fallopian tubes were preserved.

We injected the uterus with 6-unit diluted vasopressin, 50 mL in total, and then dissected the uterus longitudinally using a blade from fundus of uterus to isthmus uteri. We then continually dissected the cavum cavity. We used one dry gauze to wipe the cavum cavity, scraped the endometrium to avoid internal and external adenomyosis, and disinfected the cavum cavity with iodine complex. While inserting an index finger in the uterine cavity, the adenomyosis lesion was excised to a thickness of 3 mm of the inner myometrium on both sides. Along the serosal myometrium, the adenomyosis lesion was then excised as thin as possible on both sides. Next, we disinfected the cavum cavity again. Subsequently, the uterine cavity was sutured and closed using 3-0 absorbable suture, forming a complete uterine cavity. The next step was uterine rejoining. We sutured the serosal myometrium using 2-0 absorbable suture. At this time, to avoid creating dead space in the sutured area, a suture needle was inserted at the surface of one through the serosal surface on the original side for ligation. Most important thing is that when rejoining the uterus, the serosal myometrium on both sides should be overlapped as tightly as possible. Before closing the abdomen, we checked whether there was bleeding and flushed the abdominal cavity with 1000 mL saline. We did not use antiadhesive materials or tourniquets (Figures 2 and 3).

Three months after surgery, dysmenorrhea and menorrhagia were evaluated; TVS and MRI were checked in all patients, and pregnancy was permitted.

### 2.4. Follow-Up

Surgical efficacy was evaluated according to the severity of dysmenorrhea and hypermenorrhoea, the serum CA 125, and uterus size before and at 1, 3, 6, 12, and 24 months after surgery.

### 2.5. Statistical Analysis

Data were analysed using statistical software (SPSS, CA, USA). Results were given as mean (SD) and 95% confidence interval (CI), median (interquartile range [IQR]), or percentage, depending on the data type and distribution. Preoperative and postoperative results were compared using the using Student's* t*-test. Variables were compared among groups using repeated measures of ANOVA. All tests were 2-sided, and p < 0.05 was considered statistically significant.

## 3. Results

Between January, 2012, and December, 2014, 198 women met the inclusion criteria and underwent U-shaped myometrial excavation and modified suture approach with uterus preservation to treat diffuse adenomyosis. The average age of the patients was 36.2±8.6 years (range 26-44 years). The mean course was 7.1 years (range, 5-15 years). The mean operative time was 43.8±19.3 minutes (range, 38-76 minutes), and the mean blood loss was 113±68 mL (range, 98-205 mL). The mean weight of resected adenomyosis tissue was 608±281 g (range, 281-1480 g). Of these, 188 patients were followed up over two years, with a follow-up rate of 94.9%. Histological evaluation by the pathologist confirmed adenomyosis in all patients. All patients underwent only the previously described surgery. Patients were managed through the same clinical path commonly used for open abdominal surgery for benign disease at our hospital. Menstruation resumed in all women within 3 months after this surgery.

**Figure 1:**
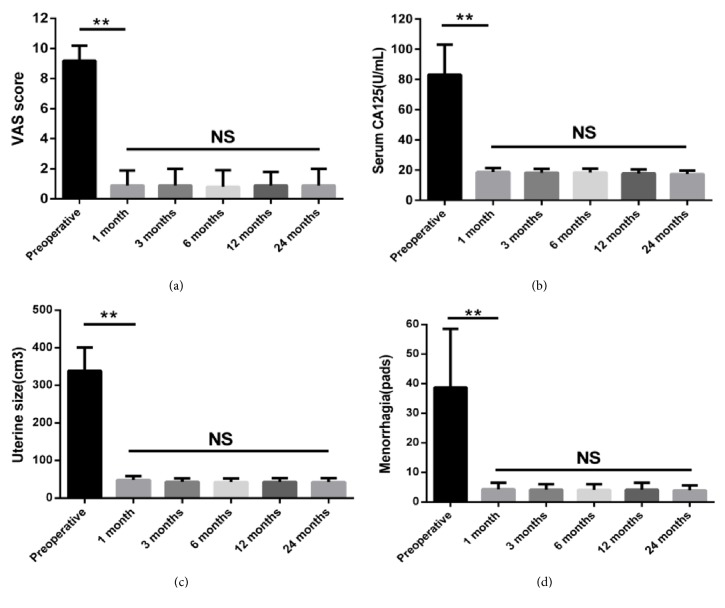
Changes in VAS scores (a), serum CA125 levels (b), uterine size (c), and menorrhagia (d) before and 1 month, 3 months, 6 months, 12 months, and 24 months after surgery.

**Figure 2:**
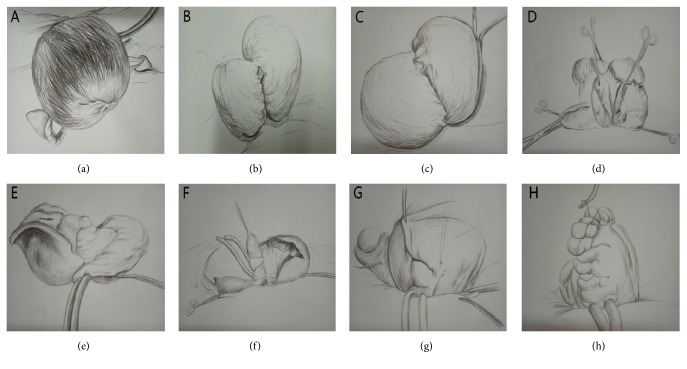
Schematic images of surgical steps of U-shaped myometrial excavation and modified suture approach with uterus preservation. (a) Uterine body. (b) Incised uterus. (c-d) U-shaped lesions resection. (e-f) Residual serosa and mucosa of uterine. (g-h) Modified serosal layer suture.

**Figure 3:**
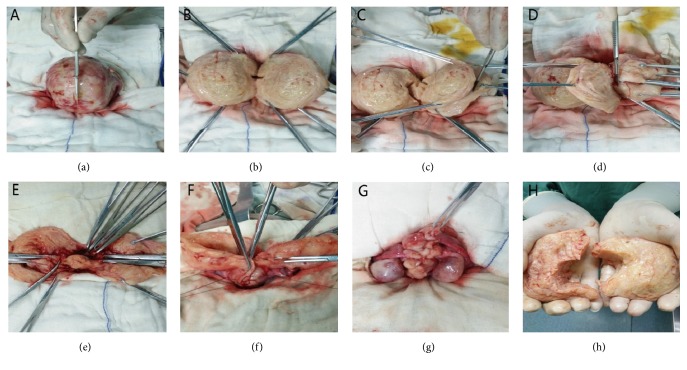
Actual images of surgical steps of U-shaped myometrial excavation and modified suture approach with uterus preservation. (a) Uterine body. (b) Incised uterus. (c-d) U-shaped lesions resection. (e) Residual serosa and mucosa of uterine. (f-g) Modified serosal layer suture. (h) Resected adenomyosis.

Dysmenorrhea, menstrual blood volume, serum CA 125 level, and uterine size were compared preoperatively and postoperatively in all patients (Table 1). As shown in the figure, the mean VAS score of dysmenorrhea, menstrual blood loss serum CA 125 level, and uterine size all significantly decreased postoperatively from preoperative baseline. However, these indexes did not distinctly change at the various postoperative follow-up time points of 1, 3, 6, 12, and 24 months (Figure 1).

All patients had less menstruation after surgery, and the majority of patients also showed marked improvement in their anaemia. Unbelievably, two of the 198 patients (1%) became pregnant and gave birth to two healthy babies. One of the two women got pregnant 13 months after surgery. Her adenomyosis lesion had been located on the anterior wall of the uterus and the maximum meridian of the uterus was 76 mm. The woman underwent caesarean section at 35 weeks and gave birth to a healthy baby. The other patient became pregnant 16 months after surgery. Unfortunately, she lost her baby at 76 days of pregnancy. At 23 months after this spontaneous abortion, she became pregnant again and gave birth to a healthy baby. The adenomyosis lesion for this woman had been located on the right posterior wall of the uterus, with maximum meridian of the uterus 66 mm.

One of the 198 patients (0.5%) had a complication of progressive anaemia and low fever after surgery. We thought the reason may be the remaining dead space in the sutured area or intraluminal haemorrhage. The ultrasound revealed a shadow of 50 mm × 30 mm × 30 mm on the right top of uterus. We gave several treatments on suspicions, and the woman finally recovered 12 days after surgery. Three months later, the ultrasound revealed that the shadow had disappeared. No remaining patient suffered complications, including postoperative incision infection, incision bleeding, or others. During the two-year follow-up, only two of 198 patients (1%) had recurrent symptoms, including dysmenorrhea and increased menstrual volume. The two patients with recurrences were at 6 and 11 months after surgery. Both eventually recovered after repeat surgery.

## 4. Discussion

Adenomyosis, a common refractory disease in women of reproductive age, is a benign lesion in which the endometrium invades the myometrium [8]. The incidence is 5-70%, greatly affecting the health of women of reproductive age [9]. Although adenomyosis is histologically benign, its clinical manifestations, including proliferation, invasion, metastasis, and high recurrence rate, always point to “malignant.” Diffuse adenomyosis is characterized by unclear boundaries between the myometrium and myometrium, the extent of the lesion and the region, and even the entire myometrium [10, 11]. Treatment of diffuse adenomyosis depends on how to safely and effectively excise unclear ranges of the lesions, including hysterectomy.

Adenomyosis showed a younger age of onset, causing patients to request preservation of the integrity of the uterus and further reproductive function [12, 13]. Radical surgery, namely, hysterectomy, is unacceptable for these patients who strongly wish to preserve their uterus. The current study focused on the problem of how to effectively treat patients with adenomyosis, protecting the uterus and reproductive function, while improving clinical symptoms.

Our initial surgical procedure for diffuse adenomyosis was to simply excise the adenomyosis lesion from the mesometrium. However, some women still had dysmenorrhea, menorrhagia, and even recrudescence of adenomyosis. At that time, they had to undergo hysterectomy. These experiences led us to develop the surgical procedure described above.

Compared with other surgery procedures [11, 14–16], the greatest advantage of this operation of U-shaped myometrial excavation and modified suture approach with uterus preservation was full consideration of the uterine anatomy. We noticed that once the uterus was excised along the median sagittal plane, the adenomyosis lesion was entirely exposed to us. We knew it was impossible to excise the entire lesion, so what we did was to effectively resect the lesion to the greatest extent possible.

In our surgical procedure, when the uterus was divided into two, the cavum cavity must be wiped clear to ensure no displacement of endometrium. On the other hand, when rejoining the uterus and suturing the serosal myometrium, both sides of the serosal myometrium must be stacked tightly together by the modified suture method to ensure no dead space in the sutured area, and the ligation must be tightly reinforced. There were two advantages to this suture: one is that it could play the role of compression and haemostasis, and the other is that it could increase the thickness of the wall of the uterus and lay the foundation for future pregnancy. Simultaneously, the uterine arteries were preserved and the blood supply of the ovaries was not affected. The greatest advantage of this operation was retaining almost complete muscularis mucosae and complete morphology of the uterus. The shape and the size of the uterus were close to those of a normal uterus 6 months after this surgery.

The symptoms, such as dysmenorrhea and menstruation, of the 198 patients greatly improved after the surgery with a low recurrence rate of 1% and complication rate of 0.5%. This was acceptable during the follow-up over two years. In our opinion, the ideal therapeutic effect of this surgical method with uterus preservation for diffuse adenomyosis derived from the almost hollowing-out of the myometrium of the diffuse uterine adenomyosis, thin uterine serosal myometrium, and muscularis mucosae, resulting in poor blood supply and atrophy of the remaining part of the adenomyosis lesion. In this way, our surgical procedure showed good outcomes and low rates of recurrence and complication.

We previously mentioned that patients were permitted to become pregnant 3 months after the surgery. However, even if a normal pregnancy is achieved, the safety remains unknown during pregnancy. Residual adenomyosis in the myometrium, combined with surgical trauma, may increase the risk of miscarriage or uterine rupture. Once the patient was pregnant, she must be carefully followed up. In the current study, there were two cases of postoperative pregnancy. One of these patients had lesions focused on the anterior wall of the uterus, while the other patient's lesions were primarily focused on the posterior wall. Therefore, for patients whose lesion was not diffuse to the entire uterus, it is possible to have baby after surgery. We must note that both women had caesarean sections at 34 or 35 weeks, because of the unbearable tension of abdominal pain and the possibility of uterine rupture. Thus, the risks during pregnancy after this surgery is high, and caesarean section should be carried out ahead of time. Despite a low postoperative pregnancy rate, our surgical procedure provided dramatic relief from dysmenorrhea and menorrhagia and improves anaemia by reducing menstrual blood loss. This is an enormously encouraging for women with severe dysmenorrhea and menorrhagia preoperatively and is one reason for the high satisfaction of postoperative patients.

Minimally invasive laparoscopic surgery is the mainstream of modern surgery; however the surgeon could not access lesions in the uterus and deep lesions are easily missed [12, 17, 18]. In addition, the extent of resection is definitely less than that of laparotomy and suture and haemostasis is also quite difficult, resulting in a relatively high postoperative recurrence rate [19]. Therefore, we do not recommend a laparoscopic approach for this procedure.

The most important benefits of our surgery are relief from dysmenorrhea and improvement of menorrhagia and anaemia, leading to high quality of physical and psychological life. Even more so, two infertile women even got pregnant after this surgery. Briefly, this surgical procedure gives hope to those who want relief from dysmenorrhea and menorrhagia and who strongly want to preserve the uterus.

## 5. Conclusion

Our surgical technique of U-shaped myometrial excavation and modified suture approach with uterus preservation provided good outcomes in women with severe symptoms of diffuse adenomyosis and should be considered as a useful treatment option for these patients.

## Figures and Tables

**Table 1:** Changes in VAS scores, serum CA125 levels, uterine size, and menorrhagia before and after surgery.

	**VAS score**	**Serum CA125** **(U/mL)**	**Uterine size** **(cm3)**	**Menorrhagia** **(pads)**
**Preoperative**	9.2±1.0	83.2+19.8	338.47±62.73	38.7±19.8
**1 month**	0.9±1.0	18.6±2.8	48.12±10.23	4.3±2.2
**3 months**	0.9±1.1	18.2±2.5	43.36±9.22	4.2±1.8
**6 months**	0.8±1.1	18.4±2.5	42.59±9.77	4.1±1.9
**12 months**	0.9±0.9	17.7±2.6	43.02±10.01	4.2±2.3
**24 months **	0.9±1.1	17.4±2.3	42.86±10.26	3.9±1.8

## Data Availability

Raw data can be provided by the corresponding author (Wang Bin) when needed.
